# Isoform Composition and Gene Expression of Thick and Thin Filament Proteins in Striated Muscles of Mice after 30-Day Space Flight

**DOI:** 10.1155/2015/104735

**Published:** 2015-01-18

**Authors:** Anna Ulanova, Yulia Gritsyna, Ivan Vikhlyantsev, Nikolay Salmov, Alexander Bobylev, Zarema Abdusalamova, Vadim Rogachevsky, Boris Shenkman, Zoya Podlubnaya

**Affiliations:** ^1^Institute of Theoretical and Experimental Biophysics, Russian Academy of Sciences, Institutskaya Street 3, Pushchino 142290, Russia; ^2^Pushchino State Institute of Natural Science, Nauki Street 3, Pushchino 142290, Russia; ^3^Dagestan State University, Gadzhieva Street 43a, Makhachkala, Republic of Dagestan 367000, Russia; ^4^Institute of Cell Biophysics, Russian Academy of Sciences, Institutskaya Street 3, Pushchino 142290, Russia; ^5^SRC, Institute for Biomedical Problems, Russian Academy of Sciences, Khoroshevskoye Street 76A, Moscow 123007, Russia

## Abstract

Changes in isoform composition, gene expression of titin and nebulin, and isoform composition of myosin heavy chains as well as changes in titin phosphorylation level in skeletal (m. gastrocnemius, m. tibialis anterior, and m. psoas) and cardiac muscles of mice were studied after a 30-day-long space flight onboard the Russian spacecraft “BION-M” number 1. A muscle fibre-type shift from slow-to-fast and a decrease in the content of titin and nebulin in the skeletal muscles of animals from “Flight” group was found. Using Pro-Q Diamond staining, an ~3-fold increase in the phosphorylation level of titin in m. gastrocnemius of mice from the “Flight” group was detected. The content of titin and its phosphorylation level in the cardiac muscle of mice from “Flight” and “Control” groups did not differ; nevertheless an increase (2.2 times) in titin gene expression in the myocardium of flight animals was found. The observed changes are discussed in the context of their role in the contractile activity of striated muscles of mice under conditions of weightlessness.

## 1. Introduction

It is known that exposure of humans or animals to real or simulated microgravity (head-down-tilt bed rest, “dry” immersion for humans, and hindlimb suspension animal models) leads to the development of changes in the sensory-motor system [1, 2]. Decreases in muscle tone and force of muscle contraction occur during short-term (up to 5 days) exposure to microgravity. Longer exposure to microgravity results in the atrophy of muscle fibers of both slow and fast types [3, 4], an increase in degradation of myosin heavy chains [5], a shift in myosin phenotype towards increase in the content of fast isoforms of heavy chains of this protein [6, 7], disruptions of sarcolemmal dystrophin [8], and disorganization of desmin and plectin which leads to Z-streaming of atrophic muscle fibers [9]. Microgravity conditions are further associated with a decrease in the content of the giant sarcomeric proteins titin (connectin) and nebulin in human [10] and animal [11–13] skeletal muscles, resulting in abnormalities in the sarcomeric structure [13] and deterioration of muscle contractile function [12, 13]. These alterations are more profound in postural, “antigravity” muscles and, to a lesser extent, in muscles involved in fast movements [3, 4].

Atrophic changes in m. extensor digitorum longus and m. soleus of rats were first observed after completion of the Cosmos 605 spacecraft mission (1973) [14]. However, according to the results of electron microscopy, no significant changes in the ultrastructure of fibers of m. soleus and m. gastrocnemius were found in rats after completion of Cosmos 605 (1973) and Cosmos 782 (1975) spacecraft missions [15]. Sarcomere lesions (eccentric contraction-like lesions: hyperextension of sarcomeres with A-band filaments pulled apart and fragmented) were detected in atrophied adductor longus and soleus muscles of rats after the Cosmos 1887, SLS-1, and SLS-2 space flights [16, 17]. Electron microscopy studies of soleus muscle fibers from pre- and postflight biopsies of four astronauts orbited for 17 days during the Life and Microgravity Sciences Spacelab Mission (1996) revealed decreased thin filament density and length [18].

Atrophic and morphological changes were observed in cardiac muscles of humans and animals after their exposure to conditions of both simulated [19] and real [20, 21] microgravity. In particular, after the COSMOS 2044 flight for 14 days, light-microscopy studies have shown an atrophy of papillary muscles in rat left cardiac ventricle [21]. Atrophic and morphological changes were also observed in the left ventricle of rat heart tissue from animals aboard the Cosmos 1887 biosatellite for 12.5 days [20, 22]. The flight animals exhibited some patchy loss of protofibrils (actin and myosin filaments) and some abnormal supercontracted myofibrils that were not seen in the controls [22]. After a 14-day simulation of gravity discharge, there was a decrease in connexin 43 (gap junction intercellular protein) content in rat myocardium [23]. According to the authors, it contributed to the development of heart arrhythmias. Also, the contractile force and velocity of cardiac muscle were shown to decrease in tail suspension rats, as compared with the control [24]. At the same time, the absence of changes in the expression of myosin heavy chains, tropomyosin, and troponin T and I isoforms was found. However, an increase in the content of cardiac troponin I fragment (cTnI) was registered in the heart of tail suspension rats. This fragment, according to the authors, is involved in functional adaptations of cardiac muscle [24]. In our studies, no changes in the content of MyBP-C, as well as *α*- and *β*-isoforms of myosin heavy chains in the myocardium of Mongolian gerbils (*Meriones unguiculatus*), were observed after their exposure to a 12-day space flight onboard the Foton-M3 biosatellite [25]. However, an increase in the N2BA/N2B titin ratio in the myocardium of gerbils of the flight group was found, which, most likely, contributed to the enhancement of the heart contractile function [26]. Recent studies revealed changes in gene expression and in the contents of beta-actin, gamma-actin, alpha-actinin-1 and 4 in membranous, and cytoplasmic protein fractions of left ventricle cardiomyocytes of mice after being exposed to a 30-day space flight onboard the BION-M1 biosatellite [27].

In the present study, we examined changes in the isoform composition and gene expression of titin (connectin), heavy chains of myosin (proteins of thick filaments), and nebulin (protein of thin filaments), as well as the titin phosphorylation level in cardiac and skeletal muscles of mice being exposed to a 30-day space flight onboard the BION-M1 biosatellite. Since recent evidence was obtained for the participation of chaperone Hsp90 in maintaining titin stability [28], a separate task in our study was to identify changes in the content and gene expression of Hsp90 in mouse striated muscles after the space flight.

## 2. Materials and Methods

### 2.1. Object for Study

Male C57BL/6N mice were purchased from the Animal Breeding Facility, Branch of Shemyakin and Ovchinnikov, Institute of Bioorganic Chemistry (Russian Academy of Sciences). Key dates of the flight experiment and the on-ground control experiment were published in 2014 [29]. Our experiments were performed on m. gastrocnemius muscle, m. tibialis anterior muscle, m. psoas muscle, and left ventricular myocardium samples obtained from male C57BL/6N mice, which were killed within 13–16.5 h after the BION-M1 biosatellite landing (the space flight lasted 30 days from 19 April up to 19 May 2013, Russia). There were five animals (*n* = 5) in this study group which was designated as group “Flight.” During the space flight, the animals were provided with paste-like feed with an energy value of 361.4 kcal/100 g of dried feed. There were five animals (*n* = 5) in the control group, which were housed in the animal breeding facility (vivarium) during the space flight of the BION-M1 biosatellite. Muscle tissue samples were immediately frozen in the liquid nitrogen and subsequently stored at −70°C. All of the experimental procedures on animals were approved by the Commission on Biomedical Ethics of the State Scientific Center of the Russian Federation, Institute for Biomedical Problems, the Russian Academy of Sciences.

### 2.2. SDS-PAGE and Immunoblot Analysis

To detect changes in the isoform composition and the content of titin (molecular mass 2000–3700 kDa) and nebulin (700 kDa), denaturing polyacrylamide gel electrophoresis was performed using the Tatsumi and Hattori technique [30] and the “Helicon” system (Russia). Muscle tissue (2 mg) was homogenized in a lysis buffer (12 mM Tris-HCl, 1.2% SDS, 10% glycerol, 2% *β*-mercaptoethanol or 75 mM DTT, 5 *μ*g/mL leupeptin, and E64, pH 6.8–7.0) as previously described [31]. In order to prevent destruction of titin at high temperatures [32], the electrophoretic sample was not boiled but incubated at +40°C for 30–40 minutes [31]. Just prior to gel electrophoresis, samples were centrifuged for 5 min at 10000 g. Analysis of MyHC isoforms was performed in 7% SDS-PAGE slab gel as described [33]. Analysis of Hsp90 was performed using the Laemmli technique in 10% SDS-PAGE slab gel [34]. Measurements of protein concentration were made by using a NanoDrop 1000 (Thermo Scientific, USA), then equal amounts of protein (5–15 *μ*g/line) were applied. Protein bands were revealed by Coomassie brilliant blue staining. The percentage of MyHC isoforms and the contents of titin and nebulin (relative to the content of myosin heavy chains) were determined by densitometry of gels by using Total Lab v.1.11 software. To determine the Hsp90 content, immunoblot analysis was conducted. Transfer of proteins to the PVDF membrane was carried out by the method of Towbin et al. [35]. Membranes were blocked overnight at 4°C with a blocking buffer (4% nonfat milk powder, TBS pH 7.4, and 0.1% Tween-20) and incubated for 2 h at room temperature with the primary monoclonal antibodies against Hsp90alpha (Proteintech Group, USA, 1 : 1000) and against Hsp90beta (antibodies were kindly provided by the Laboratory of cell cultures and cell engineering, Institute of Cell Biophysics RAS, Pushchino, Russia, 1 : 1000). As secondary antibodies, alkaline phosphatase-conjugated anti-mouse antibodies (Proteintech Group, USA, 1 : 1000) were used. Next, PVDF membranes were washed in TBS-Tween three times for 5 min and protein bands were detected by a staining solution (0.1 M Tris-HCl, 0.1 M NaCl, 0.05 M MgCl_2_, pH 9.5; with NBT/BCIP solution (Roche, Germany)). Protein bands were quantified by densitometry.

### 2.3. Determination of Titin Phosphorylation Level

Determination of titin phosphorylation level was carried out by the method described in [36] with negligible modifications. A native level of protein phosphorylation in gel was estimated with the help of fluorescent stain Pro-Q Diamond (Invitrogen) for phosphoproteins. For this purpose, gels were put into the solution containing 50% of ethanol and 10% of acetic acid for 12–18 hours and, after 30 min washing in distilled water, were stained for 1.5 hours. The stained gel was washed off in a Pro-Q Diamond phosphoprotein gel distaining solution (Invitrogen). Protein bands containing phosphate were viewed using Bio-Rad system Pharos. Then gels were stained with Coomassie Brilliant Blue G-250 and R-250 mixed in a 1 : 1 ratio for the control estimation of protein content.

### 2.4. RNA Analysis

Total RNA was extracted from 10 mg of frozen muscle samples using the Aurum Total RNA Fatty and Fibrous Tissue Kit (Bio-Rad, USA) according to the manufacturer's instructions. RNA concentration was determined at 260 nm using NanoDrop 1000 (Thermo Scientific, USA). Isolated RNA in aqueous solution was frozen at −70°C for storage.

### 2.5. Reverse Transcription

cDNA was synthesized using M-MLV reverse transcriptase (Eurogen, Russia). Reverse transcription was performed by incubating 1 *μ*g total RNA, random (dN)_10_ primer, dNTPs, DTT, 5*х* first strand buffer (280 mM Tris-HCl, pH 8.7; 375 mM KCl; 30 mM MgCl_2_), and MMLV reverse transcriptase for 60 min at 42°C.

### 2.6. Quantitative PCR Analysis

For quantitative PCR analysis, 1 *μ*L of cDNA was amplified in a 25 *μ*L SYBR Green PCR reaction mixture containing Taq DNA polymerase (Eurogen, Russia), Taq-buffer, dNTP, 0.25 *μ*M of each forward and reverse primers, and SYBR Green I as a fluorescent dye (Invitrogen). Specific primers were selected using the Vector NTI software (see Table 1). The amplification was monitored in real-time using the DT-322 amplifier (DNA-Technology, Russia). The quantity of mRNA relatively to the amount of mRNA housekeeping gene GAPDH was determined according to the method 2^−ΔΔCt^ [37].

### 2.7. Electron Microscopy

For ultrastructural analysis, muscle samples were prepared using a routine procedure. One mm thick tissue samples were dissected from m. gastrocnemius and cardiac muscle of mice from “Flight” and control groups before fixing in 3% paraformaldehyde and 2.5% glutaraldehyde dissolved in 0.1 M Na-cacodylate buffer (pH 7.2–7.4) for 2–4 h and postfixed in 1% OsO_4_ in the same buffer for 1-2 h. After dehydration in ethanol, the sections were embedded in Epon-Araldite. Semithin sections were prepared to achieve proper longitudinal orientation of muscle sarcomeres. Ultrathin sections were cut using a Leica UM6 ultramicrotome mounted on pioloform coated slot grids, stained, and analyzed with a JEOL 1200EX electron microscope.

### 2.8. Statistical Analysis

The results obtained during the experiments were statistically processed using the Mann-Whitney *U* test with the confidence levels *P* ≤ 0.01 and *P* ≤ 0.05 to evaluate the significance of differences between the groups. The data were represented as M ± SD, where M is the mean value and SD is the standard deviation.

## 3. Results

No changes in the weight of control and flight animal groups were found (Table 2). No significant changes in the weight of the myocardium and skeletal muscles of mice were also found. However, a significant decrease (*P* ≤ 0.05) in the weight of gastrocnemius and tibialis anterior muscles relative to body weight of animals of the flight group was revealed (Table 2), which indicates the development of atrophic changes in these muscles.

**Figure 1:**
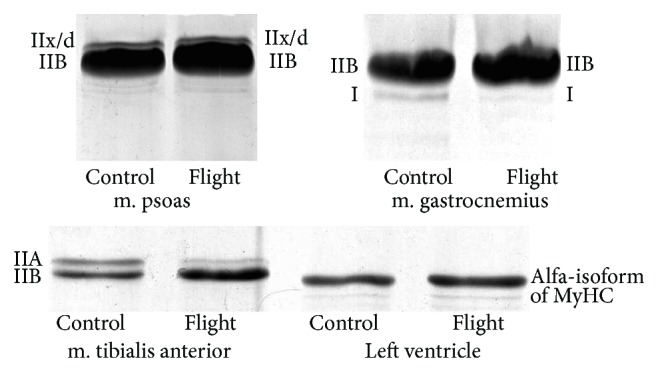
SDS-PAGE analysis of myosin heavy chains (MyHC) isoforms in striated muscles of control and flight mice. Percentage of I MyHC isoform in m. gastrocnemius of control and flight mice was 4.0 ± 1.5 and 1.55 ± 0.35 (*n* = 4), respectively. Percentage of IIA MyHC isoform in m. tibialis anterior of control and flight mice was 18.9 ± 10.6 and 6.05 ± 3.4 (*P* ≤ 0.05, *n* = 4), respectively.

Muscle disuse under microgravity conditions is known to cause slow-to-fast phenotype changes. To detect the possible changes, the expression pattern of MyHC isoforms was measured using SDS-PAGE of solubilized muscle fragments (see Figure 1). The results showed no changes in MyHC isoform contents of m. psoas, as well as in the cardiac muscle of control and flight mice (see Figure 1). Just to the contrary, the tibialis anterior muscle of flight mice showed a reduction of the IIA MyHC content, while the proportion of type IIB MyHC isoform increased under the conditions of weightlessness (see Figure 1, *P* ≤ 0.05). The decrease observed in the content of slow I MyHC isoform in m. gastrocnemius of flight group mice was however insignificant (see Figure 1). Nevertheless, the results indicate slow-to-fast phenotype changes in m. tibialis anterior and m. gastrocnemius of mice after the spaceflight.

**Figure 2:**
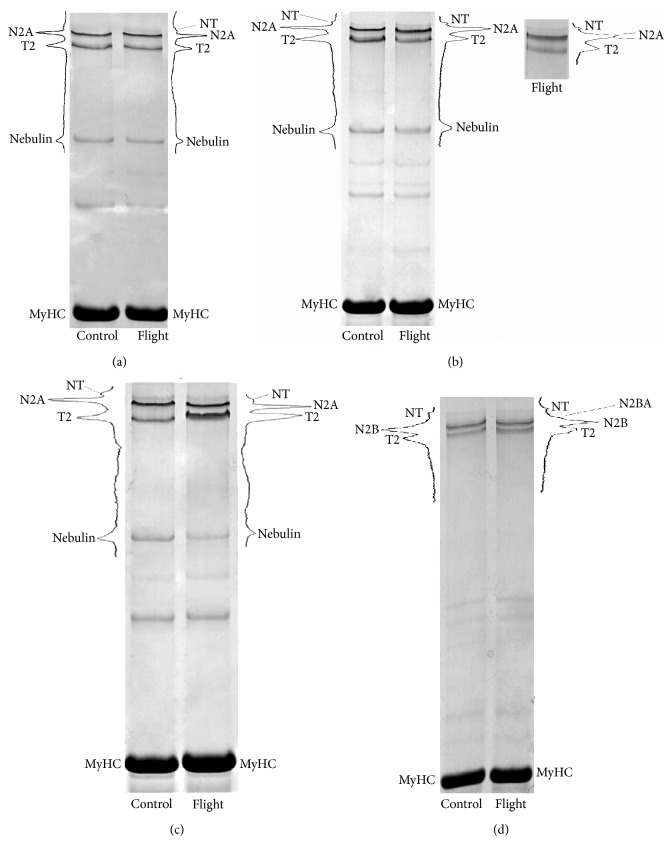
SDS-PAGE analysis of titin and nebulin expression in striated muscles of control and flight mice. (a) m. psoas; (b) m. tibialis anterior; (c), m. gastrocnemius; (d) cardiac muscle. T2 is proteolytic fragment of titin (m.w. ~2000–2100 kDa). N2B, N2BA, N2A, and NT are isoforms of intact titin (T1, m.w. ~3000–3700 kDa). NT-isoforms of titin were recently found in striated muscles of mammals [31]. The content of NT-titin in mouse striated muscles is ~10%.

**Figure 3:**
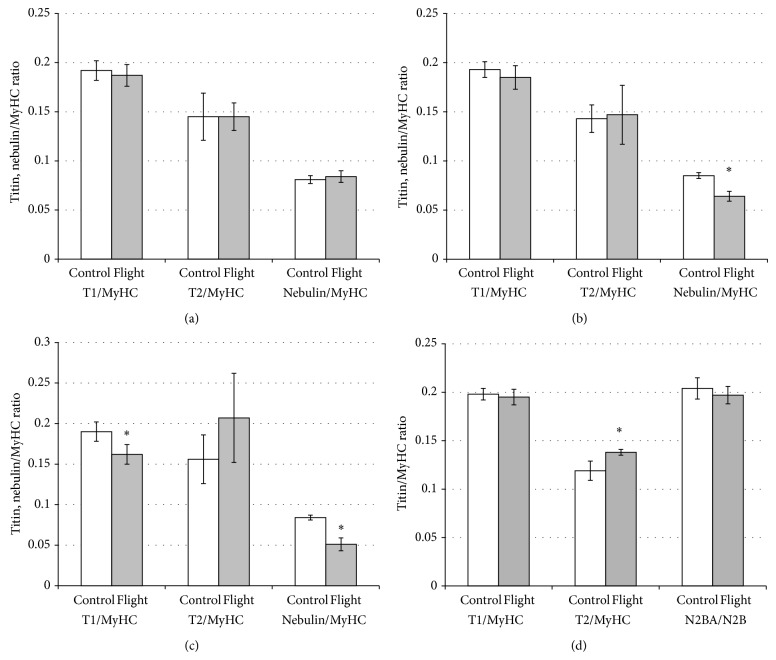
Plots of densitometric quantification of the titin and nebulin contents relative to MyHC content for control and flight striated muscles of mice. (a) m. psoas; (b) m. tibialis anterior; (c), m. gastrocnemius; (d) cardiac muscle. White plots: control, grey plots: flight. Values are means ± SD. ^*^Significant difference between control and flight mouse muscles (*P* ≤ 0.01).

Figure 2 shows the results of electrophoretic studies of the content of giant sarcomeric cytoskeletal proteins titin and nebulin in striated muscles of mice. No changes in the content of intact titin (T1) in the cardiac muscle and m. psoas, as well as nebulin in m. psoas after spaceflight, were observed (see Figures 2(a), 2(d), 3(a), and 3(d)). At the same time, a significant increase (by 16%, *P* ≤ 0.01) in the content of titin T2-proteolytic fragment in cardiac muscle of flight mice was revealed (see Figure 3(d)). A significant decrease (by 15% and by 40%, *P* ≤ 0.01) in the contents of intact titin isoforms and nebulin in m. gastrocnemius, as well as nebulin (by 25%, *P* ≤ 0.01) in the tibialis anterior muscle in mice of the “Flight” group, was observed (see Figures 2(b), 2(c), 3(b), and 3(c)). These changes in m. gastrocnemius of flight mice were accompanied by an increase by ~1.3 times in the content of the T2 (see Figures 2(c) and 3(c)). The results indicate the predominance of degradation processes in giant proteins over processes of their synthesis in these muscles. The decrease in titin content was accompanied in some cases by an appearance on the gel of a duplet band of its N2A isoform (see Figure 2(b), line 3), which also may be a consequence of increased proteolysis of this protein under microgravity conditions.

**Figure 4:**
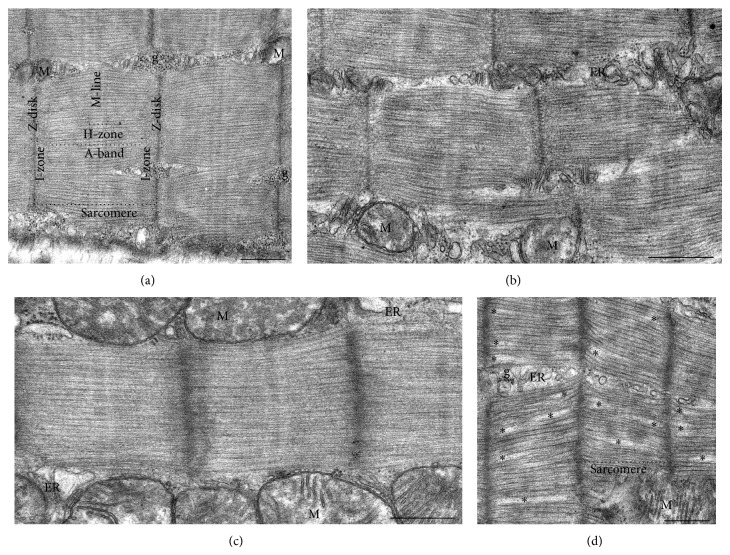
Representative views of ultrastructural organization of longitudinally sectioned sarcomeres of m. gastrocnemius (a, b) and cardiac muscle (c, d). (a, c) Control group; (b, d) flight group. The control m. gastrocnemius and cardiac muscle, as well as flight m. gastrocnemius, have similar ordered sarcomeric structures (Z-disks, M-lines, and A-, H-, and I-zones). In contrast, the flight cardiac muscle represents disorganized sarcomeric structure, with clearly visible interfilament holes (asterisks). M: mitochondria; g: glycogen granules; ER: endoplasmic reticulum. All scale bars = 500 nm.

Figure 4 shows the results of electron microscopy studies of the sarcomeric structure of m. gastrocnemius and cardiac muscle of mice. The expected disturbances in sarcomeres, in particular, hyperextension of sarcomeres with A-band filaments pulled apart and fragmented [16, 17], were not revealed in m. gastrocnemius of flight mice (see Figures 4(a) and 4(b)). Surprisingly, in the cardiac muscle of flight mice, sarcomeric structure disorders (the blurring of the boundaries of A- and I-bands of sarcomeres, an increase of interfilament spacing in A-bands) were observed (see Figures 4(c) and 4(d)), but with no decrease in titin content (see Figures 2 and 3).

**Figure 5:**
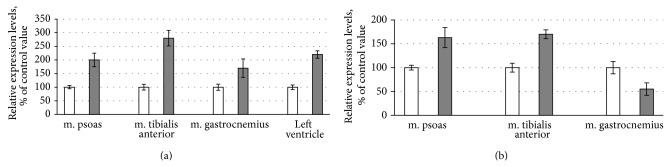
Changes in gene expression of titin (a) and nebulin (b) in mice striated muscles. White plots: control; grey plots: flight. All changes in gene expression are statistically significant (*P* ≤ 0.01).

To determine changes of gene expression of titin and nebulin, we used RT-PCR to quantify shifts in mRNA levels of their genes. The results are illustrated in Figure 5. An increase in gene expression of titin (by 1.7–2.8 times, *P* ≤ 0.01) was detected in both cardiac and skeletal muscles of flight mice (see Figure 5(a)). The gene expression of nebulin in m. psoas (by 1.63 times, *P* ≤ 0.01) and m. tibialis anterior (by 1.7 times, *P* ≤ 0.01) increased, whereas the gene expression of this protein in m. gastrocnemius decreased (by 1.83 times, *P* ≤ 0.01) in flight mice (see Figure 5(b)).

**Figure 6:**
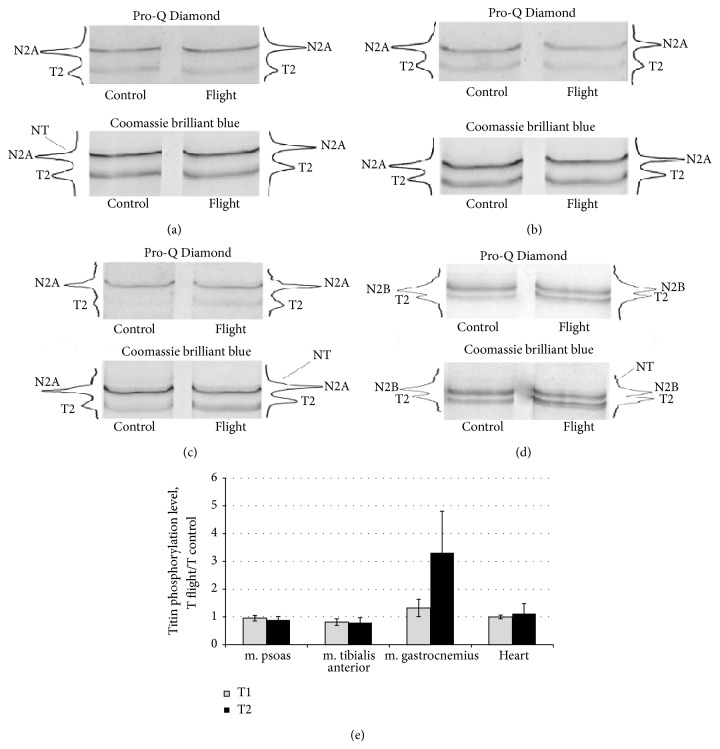
Changes in titin phosphorylation levels in striated muscles of control and flight mice. (a) m. psoas; (b) m. tibialis anterior; (c) m. gastrocnemius; (d) cardiac muscle. (e) Plots of changes in phosphorylation levels of T1 and T2 in mice striated muscles. Grey plots: T1, black plots: T2-fragment.

The ability of titin to be phosphorylated in vivo is well known [38] and the phosphorylation sites of this protein, located over the length of its molecule in the A-band (T2-titin part), I-band, and Z-disc in the sarcomere have been determined [39, 40]. We investigated the titin phosphorylation level in striated muscles of mice after their exposure to real microgravity. No significant differences in the titin phosphorylation level were revealed in m. psoas, m. tibialis anterior, and cardiac muscle of control and flight mice (see Figures 6(a), 6(b), 6(d), and 6(e)). However, the phosphorylation levels of T2 and T1 were ~3.3- and ~1.3-fold higher in m. gastrocnemius of flight mice than in the control (see Figures 6(c) and 6(e)).

**Figure 7:**
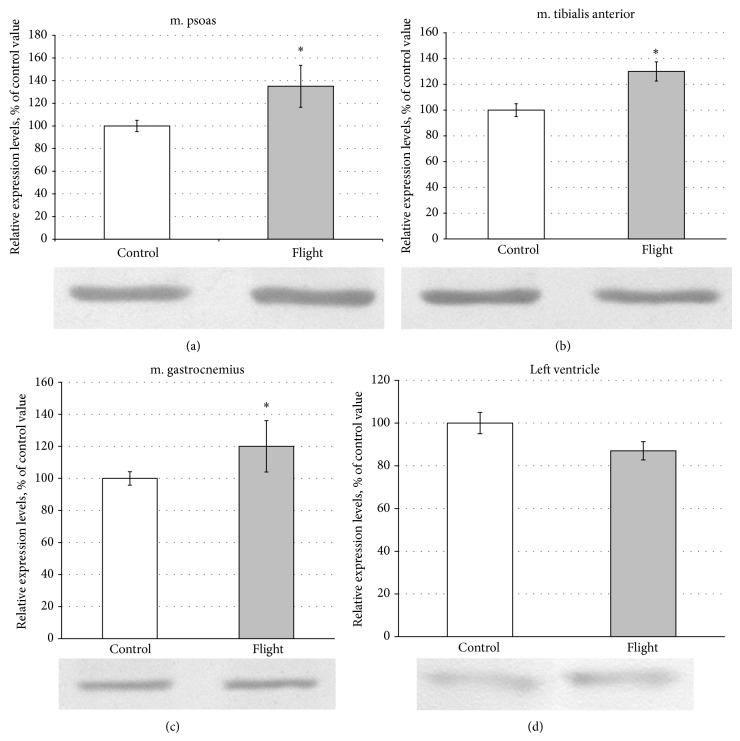
The content (immunoblot analysis data) and gene expression (plots) of Hsp90 in striated muscles of control and flight mice. White plots: control, grey plots: flight. (a, b, c) Hsp90alfa; (d) Hsp90beta. ^*^Significant difference between control and flight mouse muscles (*P* ≤ 0.01).

A separate task was to identify changes in the content and gene expression of Hsp90 in striated muscles of flight mice. The results of immunoblot analysis revealed no differences in the content of Hsp90alfa and Hsp90beta (skeletal muscles) and Hsp90beta (cardiac muscle) of control and flight mice (see Figure 7). RT-PCR results showed a slight increase (by 1.2–1.35 times, *P* ≤ 0.01) in Hsp90 (alfa and beta) gene expression in skeletal muscles and a slight decrease (by 1.15 times) in the expression of Hsp90beta gene in the myocardium of flight mice (see Figure 7).

## 4. Discussion

It is well known that disuse of muscles under conditions of both simulated and real microgravity leads to the development of atrophic changes in them [3, 41]. In particular, following 91 days of long-term exposure to real microgravity in space (MDS Mission), atrophy was evident in soleus and neck muscles of mice [41, 42]. It has been shown that hindlimb unloading causes atrophy of soleus, plantaris, gastrocnemius, and tibialis anterior muscles in mice [43, 44]. Our results showing a decrease in the weight of gastrocnemius and tibialis anterior muscles relative to body weight of flight mice (Table 2) indicate the development of atrophic changes in these muscles that are consistent with the literature data mentioned above, but contrast with data that did not reveal atrophic changes in gastrocnemius and tibialis anterior muscles of rats space flown for 14 days [45].

Skeletal muscle atrophy might be probably mainly due to activation of ubiquitin-dependent proteasome pathways. This hypothesis is consistent with the data showing increasing degradation of myosin heavy chains in association with the activation of the ubiquitin-proteasome pathway in atrophied gastrocnemius muscle of rats exposed to 16-day spaceflight (STS-90) [5], as well as with data showing an increase in the expression of MuRF-1 and atrogin-1 (MAFbx), proteins associated with the ubiquitin-proteasome system, in skeletal muscles of mice after being exposed to a 91-day space flight [41].

Atrophic changes in the skeletal muscles of flight mice led to slow-to-fast phenotype changes (see Figure 1). Our results are consistent with the slow-to-fast fibre transitions in the medial gastrocnemius and tibialis anterior muscles of rats after the 14-day flight onboard the Spacelab Life Sciences-2 (SLS-2) space shuttle [45]. However, other studies did not reveal significant changes in MyHC isoform expression in gastrocnemius and plantaris muscles of mice space flown for 11 days and 19 h (space shuttle Endeavour (STS-108/UF-1) [46] or in gastrocnemius and tibialis anterior muscles of Mongolian gerbils space flown for 12 days (Foton-M3 Mission) [47]. No reliable changes in isoform composition of MyHC in fast-twitch psoas muscle of flight mice were found in our experiments (see Figure 1). Discussing these results, it should be noted that no changes in the MyHC content of the fast-twitch EDL muscle, containing, as m. psoas do, IIx/d and IIB MyHC isoforms, were detected in mice after a 91-day space flight [41]. There is a number of data about signaling pathways, responsible for transformation of myosin phenotype in skeletal muscles under the conditions of gravitational unloading. In particular, the involvement of NF-kappaB in the slow-to-fast shift of myosin isoforms and in the development of atrophic changes in the skeletal muscles of mice during unloading has been shown [48]. And, vice versa, the expression of a calpastatin transgene slowed down the muscle wasting and obviated changes in myosin isoform expression during murine muscle disuse [49]. It is well known that the calcineurin/NFAT pathway possesses a stabilizing function counteracting the myosin phenotype transformation under gravitational unloading [50]. In another study, these authors have shown that the increase in the level of 140 and 86 kDa NFATc1 in the nucleus of m. soleus of hindlimb unloading rats is accompanied by a decrease in the percentage of fibers containing type I MyHC and an increase in the percentage of muscle fibers containing type IIA MyHC [51]. The recent data indicate the role of epigenetic modification of histones at MyHC genes in the mechanisms of slow-to-fast MyHC gene switching during unloading. In particular, it was shown that during MyHC transitions with muscle unloading, histone H3 at the type I MyHC gene becomes deacetylated in correspondence with the downregulation of that gene, while upregulation of the fast type IIX and IIB MyHCs occurs in conjunction with enhanced H3ac in those MyHC genes [52, 53]. All of these data indicate the complexity and diversity of signaling mechanisms responsible for the transformation of MyHC isoforms in skeletal muscles under conditions of gravitational unloading. Our results on changes in the content of MyHC isoforms in m. tibialis anterior and m. gastrocnemius of mice may be due to activation of different signaling pathways during space flight. To understand these signaling mechanisms, further research is needed.

Considering experimental data obtained previously [20, 21], we expected to find atrophic changes in the heart muscle of flight mice. However, such changes were not observed (Table 2). There were also no changes in the isoform composition of myosin heavy chains in the heart of flight mice (see Figure 1). In particular, the cardiac muscle of control and flight mice contained only alpha-isoform of MyHC. These results are consistent with the absence of changes in MyHC isoform composition in the heart of Mongolian gerbils after a 12-day space flight [25] and may indicate an adaptation of the heart muscle of mice to the conditions of real microgravity.

The development of atrophic changes in the skeletal muscles of animals and humans both in the simulated [10, 11, 13] and real [47] microgravity is accompanied by a decrease in the contents of giant sarcomeric proteins titin and nebulin. Our results showed no changes of titin content in m. psoas and m. tibialis anterior in flight mice (see Figures 2(a), 2(b), 3(a), and 3(b)). A significant decrease of intact titin (T1) content was found only in m. gastrocnemius of flight mice (see Figures 2(c) and 3(c)). The decrease in the content of titin in mouse gastrocnemius muscle was about two times less than that in m. soleus of humans [10] and rats [11, 13] under the conditions of simulated microgravity but did not differ from that in m. gastrocnemius, m. tibialis anterior, and m. soleus of Mongolian gerbils after the 12-day-long space flight [47]. A reliable decrease in nebulin content was found in m. gastrocnemius and m. tibialis anterior of mice after the spaceflight (see Figures 2(b), 2(c), 3(b), and 3(c)). These data contradict with the results of our earlier studies where no changes in nebulin content in m. soleus and m. tibialis anterior of gerbils have been found after the space flight [54], they agree however with the results of our model experiments in which a similar decrease in the content of this protein in m. soleus of man was revealed [10]. Titin and nebulin are known to be substrates of calcium-dependent proteases calpains [55] whose activity has been shown to increase in skeletal muscles on the first day of gravitation unloading [56]. Taking into account this evidence as well as the results showing an increase in resting [Ca^2+^] in mouse soleus muscle after hindlimb unloading [57], we suggest that the decrease in titin and nebulin content in muscles under conditions of a gravitation unloading is the consequence of heightened calcium-dependent proteolysis of these proteins by calpains. The decrease in titin and nebulin contents may also be due to reduction of expression of genes for these proteins. However, our RT-PCR results showed an increase in the expression of gene for titin in all the three skeletal muscles of flight group mice (see Figure 5(a)). This increase is consistent with the observed absence of changes in titin content in m. psoas and m. tibialis anterior of mice after space flight. An increase in the expression of gene for titin in the absence of any decrease in the content of this protein was also detected in the cardiac muscle of flight mice (see Figures 2(d), 3(d), and 5). In this situation, a reliable, although a slight, increase (by 16%, *P* ≤ 0.01) in the T2-fragment content was observed (see Figure 3(d)), which may point to an increased turnover of titin in the heart of flight mice. In m. psoas and m. tibialis anterior of flight mice, an increase in nebulin gene expression was found (see Figure 5(b)). Nevertheless, against the background of no changes in nebulin content in m. psoas, a reliable decrease in the content of this protein in m. tibialis anterior was observed (see Figure 3(b)). Decreases in the expression of nebulin gene and in the content of this protein were registered in m. gastrocnemius of mice after space flight (see Figures 2(c), 3(c), and 5). Thus, the results point to a predominance of processes of degradation of giant proteins over processes of their synthesis in m. gastrocnemius and m. tibialis anterior of flight mice.

What are the consequences of the decrease in titin and nebulin content? It was shown, that after degradation of these proteins by low doses of ionizing radiation, the ability of single skinned muscle cells to generate both passive tension in response to stretch and active tension in response to calcium was greatly reduced [58]. These effects were accompanied by axial misalignment of thick filaments [58]. In our studies, it was shown that a 7-day exposure to “dry” immersion resulted in a decrease in maximal isometric tension of skinned human soleus fibers, a decline in the myofibrillar Ca^2+^-sensitivity, and in a relative loss in titin and nebulin content [10]. There is evidence that long-term muscle disuse causes a preferential loss of titin, which is associated with changes in muscle function [13]. In particular, the Ca^2+^-sensitivity of active force decreased following 6 wk of hindlimb immobilization in the soleus muscle of rat, accompanied by a shift in the length-active force relationship to the shorter length side. Marked changes in disused sarcomeres, with shortening of thick and thin filaments responsible for altered length dependence and expansion of interfilament lattice spacing leading to a reduction in Ca^2+^-sensitivity, were also revealed [13]. A contribution to these negative changes may also be made by a decrease in the content of nebulin which plays an important role in the regulation of thin filament length [59]. Most probably, it has been just a decrease in nebulin content, which might lead to a decrease in length of thin filaments of soleus muscle fibers in four astronauts orbited for 17 days during the Life and Microgravity Sciences Spacelab Mission (1996) [18]. Taking into account the evidence from recent studies that nebulin may also control muscle contraction at the level of individual cross-bridges [59], it is reasonably safe to state that a loss of this protein, as well as titin will cause disturbances in the contractility of m. gastrocnemius and m. tibialis anterior of flight mice. A negative contribution to the decrease in the contractility of m. gastrocnemius may also be made by the increased titin phosphorylation level, detected in this muscle of flight mice (see Figures 6(c) and 6(e)). This conclusion is based on our in vitro studies pointing to that an increase in the phosphorylation of T2 of titin decreases the activating effect of this protein on the actin-activating ATPase activity of reconstructed filaments of myosin [26, 60]. The results of these experiments, similar as the evidence for the binding of titin Fn3 domains to the sub-fragment-1 of myosin [61], may point to the involvement of titin in regulation of muscle contraction at the level of individual cross-bridges.

The detected decrease in titin and nebulin contents in m. gastrocnemius of mice after a 30-day space flight may cause disturbances in the sarcomeric structure [58]. This proposal is also based on the data pointing to that muscle atrophy occurs in microgravity, whereas sarcomere lesions are postflight phenomena [17]. In particular, sarcomere eccentric contraction-like lesions (hyperextension of sarcomeres with A-band filaments pulled apart and fragmented) were detected in rat adductor longus myofibers 4.5 h after landing; these lesions were absent in flight [17]. Because mice in our experiment were killed within 13–16.5 h after landing of BION-M1 biosatellite, sarcomere lesions were to be expected. The results of our electron-microscopy studies, however, revealed no disturbances in the sarcomeric structure of m. gastrocnemius of flight mice (see Figures 4(a) and 4(b)). Surprisingly, disruptions of the sarcomeric structure were observed in the cardiac muscle of flight mice (see Figures 4(c) and 4(d)). It should be of interest to note that similar disturbances in the sarcomeric structure were observed in rat myocardium after hypoxical preconditioning and ischemia-reperfusion of the isolated heart [62]. Disruptions that we observed in the sarcomeric structure of mouse cardiac muscle are, most likely, caused by the stress during lowering and landing of the spacecraft. Further studies are required to determine whether these changes were of adaptation or of pathologic character.

The results of recent studies revealed a significant role of chaperone Hsp90 in maintaining titin stability [28]. Taking into account this evidence, we aimed to study changes in gene expression and in Hsp90 content in cardiac and skeletal muscles of mice after the spaceflight. Possibly, any contribution to the decrease of titin content, we observed in m. gastrocnemius of mice, could also be due to a decrease in Hsp90 content. But this proposal lacked any support. In particular, on a slight (1.2-fold) increase in the expression of genes Hsp90alfa and Hsp90beta, no changes in the content of these proteins in m. gastrocnemius of flight mice were found (see Figure 7). Similar results were obtained in m. psoas and m. tibialis anterior of mice (see Figure 7). Since, according to the results of our immunoblot analysis, the Hsp90alfa isoform was not revealed in the cardiac muscle of mice, we investigated the content of beta-isoform of this protein. The results revealed no changes in Hsp90beta content on a slight (1.15-fold) decrease in the expression of its gene in the cardiac muscle of flight mice (see Figure 7). Thus, no alterations were observed in the content and gene expression of Hsp90 in cross-striated muscles of mice after their 30-day spaceflight. It is believed that calcium-dependent proteolysis of giant proteins by calpain proteases is the main molecular mechanism responsible for their low content in the m. gastrocnemius and m. tibialis anterior of mice after their 30-day spaceflight.

## Figures and Tables

**Table 1:** Primers used for qRT-PCR study.

Gene description	Forward primer	Reverse primer	Accession number GeneBank	Product size, bp
GAPDH	5′-CTACACTGAGGACCAGGTTG-3′	5′-AAGGTGGAAGAGTGGGAGTT-3′	GenBank: GU214026.1	60
Hsp90aa1	5′-AAATCCGTTACGAGAGCCTG-3′	5′-AATGGTCAGGGTTCGGTCCT-3′	NM_010480.5	101
N2B	5′-GAACGAATCCAGAGCCAGAC-3′	5′-TTTCCCACAACCCTGACTCT-3′	NM_028004.2	68
Neb	5′-GCAAGACTACAGGGAGTGGT-3′	5′-TCTGATCGCTGGCATATTCC-3′	NM_010889.1	101
N2A	5′-GGAAATATGTCTGTCAAGCC-3′	5′-GCAGAACACCTTTGTATGCC-3′	NM_011652.3	52
Hsp90ab1	5′-AAGGAACGGGAGAAGGAGAT-3′	5′-TCCTCCTTATCTTCCTCCTC-3′	NM_08302.3	77

**Table 2:** Weight ratio of gastrocnemius, tibialis anterior muscles, and heart to body in control and flight mice.

Group	Animal weight, g	Myocardium weight, mg	Weight of m. gastrocnemius med., mg	Weight of m. tibialis anterior, mg	Ratio of myocardium weight to animal body weight, mg/g	Ratio of weight of m. gastrocnemius to animal body weight, mg/g	Ratio of weight of m. tibialis to animal body weight, mg/g
Control, *n* = 5	27.8 ± 1.0	167.4 ± 23.8	85.5 ± 5.7	40.6 ± 5.1	6.03 ± 0.84	3.08 ± 0.24	1.46 ± 0.19
Flight, *n* = 5	29.3 ± 2.2	179.1 ± 7.5	76.8 ± 7.8	35.7 ± 5.1	6.13 ± 0.28	2.62 ± 0.25^*^	1.22 ± 0.15^*^

^*^ *P* ≤ 0.05. Psoas muscle was not weighed in these experiments because of methodical difficulties of isolation of the whole muscle.
